# Reproduction numbers of Andes virus vary according to social setting

**DOI:** 10.1016/j.nmni.2026.101797

**Published:** 2026-06-18

**Authors:** Yuri Amemiya, Hiroshi Nishiura

**Affiliations:** Graduate School of Medicine, Kyoto University, Yoshidakonoecho, Sakyoku, Kyoto, 6068501, Japan

**Keywords:** Hantavirus, Hantavirus pulmonary syndrome, Basic reproduction number, Transmissibility, Epidemiology

Dear Editor,

Andes virus (ANDV) is a South American orthohantavirus responsible for hantavirus pulmonary syndrome, mainly in Chile and Argentina. While hantaviruses typically spread from rodent reservoirs (i.e., the long-tailed pygmy rice rat), ANDV can cause human-to-human transmission [1]. An outbreak of ANDV on a cruise ship departing from Argentina on 1 April 2026 [2] highlighted the urgent need to understand the transmission dynamics. Although the overall transmission potential of ANDV is considered to be limited with the basic reproduction number (*R*_0_) at 0.24, secondary transmission is highly variable, sometimes involving superspreading events (SSEs). Identifying factors that drive heterogeneous transmission is crucial for efficient and targeted control of the outbreak. Here we aimed to estimate the reproduction numbers by social setting.

We systematically collected dataset of documented human-to-human transmission of ANDV [3]. We reviewed eight published studies and one official report of the World Health Organization from 1993 to 2026, including three reports from Chile, five from Argentina, and one from a cruise ship in 2026 [4]. From the transmission trees reported in the reviewed studies, we extracted the number of secondary cases generated by single primary case, and hereafter the distribution is referred to as the offspring distribution, stratified by social setting. We assumed that the number of secondary cases generated by a primary case in setting *s* (i.e., in household, hospital, or others) followed a negative binomial distribution with mean $R_{s}$ and dispersion parameter *k* assumed as common across setting. The probability of observing x secondary cases is described by the offspring distribution $f\left(x|k,R_{s}\right)$. The likelihood was constructed from two components: (i) the zero-truncated negative binomial distribution among primary cases that generated at least one secondary case, $f\left(x_{i}|k,R_{s}\right)/\left(1-p_{0,s}\right)$ which was normalized by $1-p_{0,s}$, where $p_{0,s}$ is the probability of zero secondary case in a setting *s* [5], and (ii) a Poisson distribution for cases that generated no secondary case. Within each setting *s*, the ratio of primary cases without any secondary case to other primary cases that produced at least one secondary case is $p_{0,s}/\left(1-p_{0,s}\right)$. Multiplying the ratio to *n*_*s*_, the number of cases generating one or more secondary cases, we obtained the expected number of cases without any secondary case. Summing up the expected counts of zero transmission over social settings *s* yielded the total number of zero events *λ*, written as $\sum_{s}n_{s}\;p_{0,s}/\left(1-p_{0,s}\right)$. The observed number of cases that generate no secondary case, $Z_{obs}$, was assumed to follow a Poisson distribution with mean λ. The total likelihood function was described by:

(1) $$L\left(k,R_{s}\right)=\left[\prod_{s}\prod_{i=1}^{n_{s}}\frac{f\left(x_{i}|k,R_{s}\right)}{1-p_{0,s}}\right]\frac{\lambda^{Z_{obs}}\;e^{-\lambda }}{Z_{obs}!}$$

Using equation (1), we jointly estimated *R*_s_ and *k* employing a maximum likelihood method with 95% confidence intervals (CI) computed by parametric bootstrapping.

A total of 462 human cases were reported, and we excluded 17 cases that did not allow us to identify transmission tree in each cluster. Consequently, 445 cases were included in the analysis. Of the 445 cases, 55 cases (10.1%) had at least one secondary case. Of these, 41 cases caused secondary transmission in households, 6 cases in hospital (nosocomial transmission), and 7 in other settings including 2 during car trip, and one each for a bus trip, a workplace, a party, a wake (funeral), and on the cruise ship.

The reproduction number *R*_s_ for each setting s were estimated at 0.13 (95% CI: 0.09, 0.35) in households, 0.33 (95% CI: 0.01, 1.27) in hospitals, and 2.17 (95% CI: 0.37, 6.90) in other settings, respectively. The dispersion parameter *k* was estimated at 0.29 (95% CI: 0.16, 3.45). Fig. 1 compares the observed and predicted offspring distribution by social setting.

**Fig. 1:**
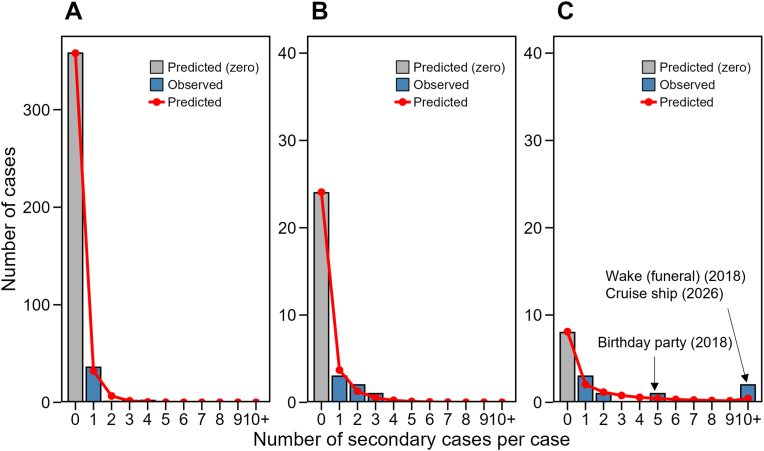
The distributions of the number of secondary cases produced by single primary case for Andes virus, 1993-2026, by social setting Each panel shows the offspring distribution that was attributed to a specific social setting: panel A for households, panel B for hospitals, and panel C for other settings. Note that the vertical axis scale is different by panel. Blue bar shows the observed number of cases that produced specified number of secondary cases, and gray bar shows the model-predicted number of primary cases that did not produce any secondary case. The red line and point show the predicted counts derived from the fitted model.

Reproduction numbers *R*_s_ in households and hospitals remained below the value of one, but the expected value of *R*_s_ in other settings was above one. Other settings included closed and crowded indoor spaces (e.g. party and cruise ships) with a large number of susceptibles. Our findings lead us to suspect that prolonged close contact in poorly ventilated and crowded environment could potentially elevate the risk of transmission. That is, SSEs might be partly determined by environmental and social conditions rather than virus or host characteristics.

While the hypothesized explanation needs to be validated, our findings offer an important public health implication. Focused interventions on specific confined spaces could efficiently help avoid and contain ANDV outbreak.

## CRediT authorship contribution statement

**Yuri Amemiya:** Conceptualization, Data curation, Formal analysis, Investigation, Methodology, Visualization, Writing – original draft, Writing – review & editing. **Hiroshi Nishiura:** Conceptualization, Investigation, Methodology, Validation, Writing – original draft, Writing – review & editing.

## Declaration of competing interest

The authors declare that they have no known competing financial interests or personal relationships that could have appeared to influence the work reported in this paper.
